# Gold Binding by Native and Chemically Modified Hops Biomasses

**DOI:** 10.1155/BCA.2005.29

**Published:** 2005

**Authors:** M. Laura López, J. L. Gardea-Torresdey, J. R. Peralta-Videa, G. de la Rosa, V. Armendáriz, I. Herrera, H. Troiani, J. Henning

**Affiliations:** 1 Envtronmental Science and Engineering Ph.D. Program University of Texas at El Paso 500 W. University Ave. El Paso TX 79968 USA; 2 Chemistry Department University of Texas at El Paso 500 W. University Ave. El Paso TX 79968 USA; 3 Department of Chemical Engineering University of Texas at Austin Austin TX 78712-1062 USA; 4 Department of Crop and Soil Science United States Department of Agriculture—Agricultural Research Service Oregon State University Corvallis OR 973311 USA

**Keywords:** Hops, chemical modification, Au nanoparticles, metal binding, Au recovery

## Abstract

Heavy metals from mining, smelting operations and other industrial processing facilities pollute wastewaters worldwide. Extraction of metals from industrial effluents has been widely studied due to the economic advantages and the relative ease of technical implementation. Consequently, the search for new and improved methodologies for the recovery of gold has increased. In this particular research, the use of cone hops biomass (*Humulus lupulus*) was investigated as a new option for gold recovery. The results showed that the gold binding to native hops biomass was pH dependent from pH 2 to pH 6, with a maximum percentage binding at pH 3. Time dependency studies demonstrated that Au(III) binding to native and modified cone hops biomasses was found to be time independent at pH 2 while at pH 5, it was time dependent. Capacity experiments demonstrated that at pH 2, esterified hops biomass bound 33.4 mg Au/g of biomass, while native and hydrolyzed hops biomasses bound 28.2 and 12.0 mg Au/g of biomass, respectively. However, at pH 5 the binding capacities were 38.9, 37.8 and 11.4 mg of Au per gram of native, esterified and hydrolyzed hops biomasses, respectively.


### Gold Binding by Native and Chemically Modified Hops Biomasses

M. Laura López[2], J.L. Gardea-Torresdey* [1,2], J.R. Peralta-Videa[2], G. de la Rosa[1], V. Armendáriz[2], I. Herrera[2], H. Troiani[3], J. Henning[4]

*[1]Environmental Science and Engineering Ph.D. Program and [2]Chemistry Department, University of Texas at El Paso, 500 W. University Ave. El Paso, TX 79968; [3]Department of Chemical Engineering, University of Texas at Austin, Austin, TX 78712-1062*
*[4]Department of Crop and Soil Science, United States Department of Agriculture–Agricultural Research Service, Oregon State University, Corvallis, OR 973311, USA*

### GRAPHICAL ABSTRACT

Traditional methods for gold recovery from wastewaters use chemical compounds that release toxic by-products to the environment. Experiments demonstrated that hop biomass could be considered as an environmentally friendly method for gold recovery from aqueous solutions. Almost 100% of Au(III) ions were bound to esterified biomass at any pH studied.

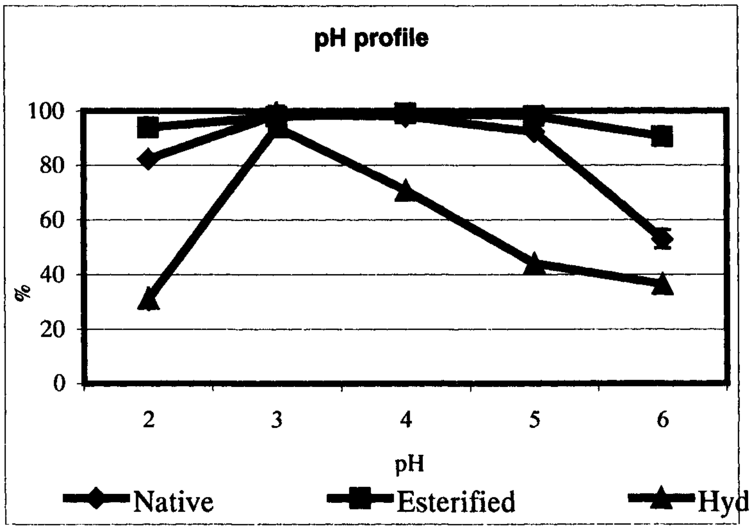

* Corresponding author: Phone (915) 747-5359; Fax (915) 747-5748 E-mail: jgardea@utep.edu

### ABSTRACT

Heavy metals from mining, smelting operations and other industrial processing facilities pollute wastewaters worldwide. Extraction of metals from industrial effluents has been widely studied due to the economic advantages and the relative ease of technical implementation. Consequently, the search for new and improved methodologies for the recovery of gold has increased. In this particular research, the use of cone hops biomass (*Humulus lupulus*) was investigated as a new option for gold recovery. The results showed that the gold binding to native hops biomass was pH dependent from pH 2 to pH 6, with a maximum percentage binding at pH 3. Time dependency studies demonstrated that Au(III) binding to native and modified cone hops biomasses was found to be time independent at pH 2 while at pH 5, it was time dependent. Capacity experiments demonstrated that at pH 2, esterified hops biomass bound 33.4 mg Au/g of biomass, while native and hydrolyzed hops biomasses bound 28.2 and 12.0 mg Au/g of biomass, respectively. However, at pH 5 the binding capacities were 38.9, 37.8 and 11.4 mg of Au per gram of native, esterified and hydrolyzed hops biomasses, respectively.

**Key Words:** Hops, chemical modification, Au nanoparticles, metal binding, Au recovery.

### INTRODUCTION

Cyanidation, smelting, ion exchange and electrolysis are different techniques widely used in mineral industries to recover gold. However, such methods are usually expensive and generate toxic chemical residues potentially harmful to the environment /1,2/. Therefore, it is necessary to explore new methodologies that will allow the recovery of gold from aqueous solutions.

Biosorption uses biological materials or wastes from other industrial processes to remove heavy metals from wastewaters /3,4/. The metal adsorbing properties depend on the type of biomass, the specific metal present in the solution, the biomass preparation process, and the chemical environment of the solutions /5,6/. Also, the metal concentration plays an important role in the heavy metal binding to the biomass /7/.

There are different mechanisms that have been postulated for adsorption processes such as chemisorption (which involves an ion exchange mechanism), complexation, coordination, chelation, and precipitation, among others /8/. Moreover, during the metal adsorption process, chemical transformation of metal ions can occur. For example, oxidation/reduction reactions usually take place in the process /9,10/.

Metals tend to modify the configuration of biological structures to form complexes or to block functional groups. Biomass cell walls have a high content of amino acids and polysaccharides, which have different functional groups able to bind metal ions /11/. Proteins such as cysteine form complexes with metal ions because they possess chemical coordination sites such as sulfhydryl, amino and carboxylate groups /12/. Other factors such as the porosity and density of biological materials contribute to the uptake of metals /13/. Recovery of metal ions from wastewaters using plant biomass could be considered a cost-effective technique /14/. As for the interaction of gold nanostructures with biological systems, some investigators have demonstrated that inactivated biomass of agricultural wastes of *Medicago sativa* (alfalfa) was able to remove Au(III) from aqueous solutions /15/. In addition, a subsequent reduction of Au(III) to Au(0) was observed. Based on such information, this study was performed with the specific aim of exploring the ability of hops biomass to adsorb Au(III) from aqueous solutions.

Different chemical modifications of hops biomass were performed in order to determine possible effects in the adsorption of Au(III). In this study, we also report on pH profile experiments as well as time dependency and capacity studies conducted with native and chemically modified hops biomasses.

### MATERIALS AND METHODS

### Hops collection

Hops biomass used for these experiments (from cone belt, harvested portion of the plant) was obtained from the United States Department of Agriculture-Agricultural Research Services (USDA-ARS) Hops Research Farm located outside Corvallis, Oregon (USA). Plants were allowed to grow to cone maturity and then cut down. The cones were separated from the rest of the plant and washed with deionized (DI) water in order to remove dirt and debris. Cone samples were then allowed to air dry for a week, dried in a forced air dryer set at 65°C, and ground with a Wiley-Mill miller. The resulting powder was passed through a 0.149 mm (100-mesh) sieve to achieve a uniform particle size.

### pH profile studies for Au(III) binding

Batch experiments were performed in order to determine the optimum pH at which Au(III) binds to hops biomass. Five samples of 500 mg each of native cone hops biomass were washed twice with 0.01M HCl in order to remove any soluble material that might interact with Au ions. This biomass was washed three times with DI water and centrifuged at 3000 rpm in a Marathon 6K Fisher Scientific Centrifuge. Washings were collected in a beaker, heated to boil, dried and then weighed to account for any loss of biomass. Each biomass sample was re-suspended in 100 mL of DI water in order to obtain a 5 mg/mL concentration. The samples were adjusted to either pH 2, 3, 4, 5 or 6 by adding either NaOH or HCl at different concentrations. Aliquots of 4 mL from each pH value were transferred to clean 5 mL test tubes and centrifuged at 3000 rpm for 5 minutes. The supernatants were separated and the pellets were saved to perform the pH profile experiments. Five volumes of 50 mL each of a 0.1mM Au(III) solution prepared from $KAuCl_4$ were also adjusted to pH 2, 3, 4, 5, and 6 as previously described in the literature /16/. Aliquots of 4 mL of Au(III) solution at the different pH values were transferred to the test tubes containing the biomass pellets at the respective pH. Samples were equilibrated for one hour on a rocker and subsequently centrifuged. The supernatants were then transferred to clean test tubes and metal analysis for Au was performed using a flame atomic absorption spectrometer (FAAS) (Perkin Elmer model 3110 with deuterium background subtraction). This process was repeated for esterified and hydrolyzed hops biomasses. Each treatment was replicated three times for Quality Control/Quality Assurance (QC/QA) purposes.

### Time dependency studies for Au(III) binding to cone hops biomass.

Time dependency experiments were performed using native, esterified and hydrolyzed cone hops biomasses in order to determine the optimum time for Au(III) binding to the biomass. The modification procedure will be explained in the next section. For this purpose, 500 mg of each biomass were washed as indicated in the pH profile section. Later, each biomass was re-suspended in 100 mL of DI water in order to obtain a concentration of 5 mg/mL. The biomass suspensions were adjusted to either pH 2 or pH 5 with HCl and NaOH solutions at different concentrations, as previously described /16/. These pH values were studied in order to determine any enhancement on Au(III) binding. Aliquots of 4 mL of the biomass suspension were transferred in two batches of 18 clean test tubes each, centrifuged, and the supernatants were separated. A solution of 0.1mM of Au(III) was prepared and adjusted to either pH 2 or pH 5. Aliquots of 4 mL were transferred to the respective pH biomass pellets and allowed to react for 0, 5, 10, 15, 30 or 60 minutes. After the reaction occurred at the appropriate interval of time, the test tubes were centrifuged and the supernatants transferred to clean test tubes to determine the Au concentration using a FAAS. Studies were performed in triplicate for each native, esterified and hydrolyzed hops biomasses.

### Chemical modification of hops biomass

The chemical modification of hops biomass was performed following the same procedure previously reported in the literature /17/. Such experiments were carefully conducted since the selective modification of the functional groups present in the hops biomass provides information about the nature of the metal binding sites.

### Esterification

Esterification of hops biomass was performed following the same procedure previously reported /17/. This reaction was done in order to block carboxyl groups on native hops biomass. The biomass was washed twice with 0.01M hydrochloric acid to remove any debris and three times with deionized water. All the supernatants were collected and dried to account for any loss of biomass during washings. After that, the biomass was resuspended in an acidic methanol solution (in 0.1M HCl), stirred, and heated at 60°C for 48 hours. Biomass pellets were made by centrifugation. The supernatants were collected and dried to account for any loss of biomass. The biomass was washed three times with deionized water, centrifuged and lyophilized for further metal binding experiments. The basic chemical reaction is represented below:

$$\text{Biomass----}\overset{O}{\overset{\|}{C}}\text{---- OH} + CH_3\,OH \xrightarrow{HCl} \text{Biomass----}\overset{O}{\overset{\|}{C}}\text{-----O----}CH_3 + H_2O$$

### Hydrolysis

The hydrolysis of hops biomass was performed in order to increase the amount of carboxyl groups in the biomass. The biomass was washed twice with 0.01M HCl and three times with DI water as described before. All supernatants were collected and dried in order to account for any biomass loss. The biomass was then reacted with 100 mL of 0.1M NaOH as previously described /17/ for one hour and was later washed three times with DI water, centrifuged, lyophilized, and saved for subsequent metal binding experiments. The basic reaction for hydrolysis of hops biomass is represented below:

$$\text{Biomass----COO–CH}_3 \quad + \quad \text{NaOH} \longrightarrow \text{Biomass---- COO}^- + \text{CH}_3\text{OH} + \text{Na}^+$$

### Gold binding capacity experiments

Binding capacity experiments were performed in order to determine the capability of the native and chemically modified hops biomasses to bind Au(III) ions from an aqueous solution. In this experiment, samples of 150 mg of each hops biomass were washed twice with 0.01M HCl and three times with DI water. Each biomass sample was re-suspended in 30 mL of DI water in order to obtain a concentration of 5 mg/mL and then adjusted to either pH 2 or pH 5. Aliquots of 4 mL from each biomass suspension were transferred to clean test tubes, centrifuged for 5 minutes at 3000 rpm, and supernatants discarded. Aliquots of 4 mL of a 0.3 mM Au(III) solution previously adjusted to either pH 2 or pH 5 were transferred to the tubes containing the biomass pellets at the corresponding pH. A volume of 4 mL of Au(III) was set aside as the control. Subsequently, all the tubes were equilibrated on a rocker to allow the reaction between the biomass and the Au(III) solution. After 15 min, the test tubes were centrifuged, the pellets saved, and the supernatants were analyzed for Au content using FAAS. The same procedure was repeated 9 times on the previously reacted pellets in order to obtain the biomass saturation. Each treatment was repeated three times for statistical purposes. Standards used in the FAAS analysis were prepared by dilution of the original 1000 ppm solution using 5% $HNO_3$. Au-saturated biomass pellets were saved for further Au recovery experiments.

### Stripping of gold

Previous reports indicated that thiourea has the ability to remove gold from the biomass /18/. Therefore, the gold-laden biomass pellets resulting from the binding capacity experiments were reacted with different concentrations of thiourea (in 0.2M HCl) in order to determine the best concentration of the stripping agent needed to remove the Au bound to hop biomass. The following concentrations of thiourea were tested: 0.1, 0.2, 0.4, and 0.6 M. Each concentration was also adjusted to either pH 2 or 5. The thiourea solutions were added to different pellets at the respective pH and allowed to react for 15 minutes under rocking before centrifugation. Each treatment was repeated three times. Supernatants were transferred to clean test tubes and diluted with 5% $HNO_3$ for metal analysis under FAAS.

### Metal Analyses

Gold content in all of the experiments was determined using FAAS. The readings were performed at a wavelength of 242.8 nm, a lamp current of 12 mA, and a slit of 0.7 mm. An impact bead was used to improve the instrument sensitivity. The samples were diluted with a 5% $HNO_3$ solution. Four standards were used in order to obtain the calibration curve and the correlation coefficient ($r^2$) obtained was 0.99 or better. The difference between the concentration present in the control and the concentration of Au in the samples after reaction was assumed to be the metal bound to the hops biomass. All samples were analyzed in triplicate. The mean values and 95% confidence intervals were calculated.

### RESULTS AND DISCUSSION

### pH profile experiments

Figure 1 shows the results for the pH profile experiments for Au(III) binding to hops biomass in a pH range from 2 to 6. In this figure, Au(III) binding to esterified hops biomass is shown to be pH independent. On the contrary, the metal binding to native and hydrolyzed hops biomasses showed a Gaussian curve with a maximum at pH 3 and 4 for native and a maximum at pH 3 for hydrolyzed hops biomass. The results reported herein were similar to those obtained by the exposure of alfalfa biomass to solutions containing Au(III) /15/, where the authors found that Au(III) binds to native alfalfa biomass in a pH independent manner. In those studies, they also reported that modified tissues of alfalfa biomass showed an enhancement or a reduction of the metal binding depending on the chemical modification carried out. In the hydrolyzed hops biomass the amount of carboxyl groups is increased and as pH increases the availability of carboxylate moieties also increases. Since Au(III) is present as the anion tetrachloroaurate ($AuCl_4^-$) and because of the repulsion between the negative charges in the carboxylate groups and the anion, Au binding decreases as the pH increases. $AuCl_4^-$ binding to carboxylate groups is expected to decrease with increasing pH. However, the actual binding mechanism has not yet been determined and other factors might be involved. Figure 1 also shows that the percentage of Au(III) bound to native hops biomass is higher than the one obtained from the hydrolyzed hops biomass. Perhaps the amount of carboxyl groups is smaller in native than in hydrolyzed biomass, then, Au(III) binds better to native hops biomass. In Figure 1, it is also apparent that at pH 2, the percentage of Au(III) bound to hydrolyzed biomass is much lower compared to native and esterified biomasses. If Au(III) was present as an anion, an electrostatic interaction between the protonated carboxyl group and the Au(III) anion may lead to an increase in percentage binding, which was not observed. It is possible that tetrachloroaurate behaves differently, depending on the pH of the solution. We propose that at pH 2, the tetrachloroaurate anion releases chloride species faster than at pH 5, which contribute for the reduction of Au(III) to Au(0). Thus, at pH 2, the repulsion between positively charged species (protonated carboxylates and $AuC1_2^+$) leads to a reduction in Au binding. Since native and esterified hops biomasses do not have as many carboxylate groups as hydrolyzed biomass, the repulsion is diminished allowing for Au binding to other centers different from carboxyl moieties. If the release of chloride ions is slower at pH 5, the repulsion between the carboxylate species and the anion tetrachloroaurate results in a reduction of Au

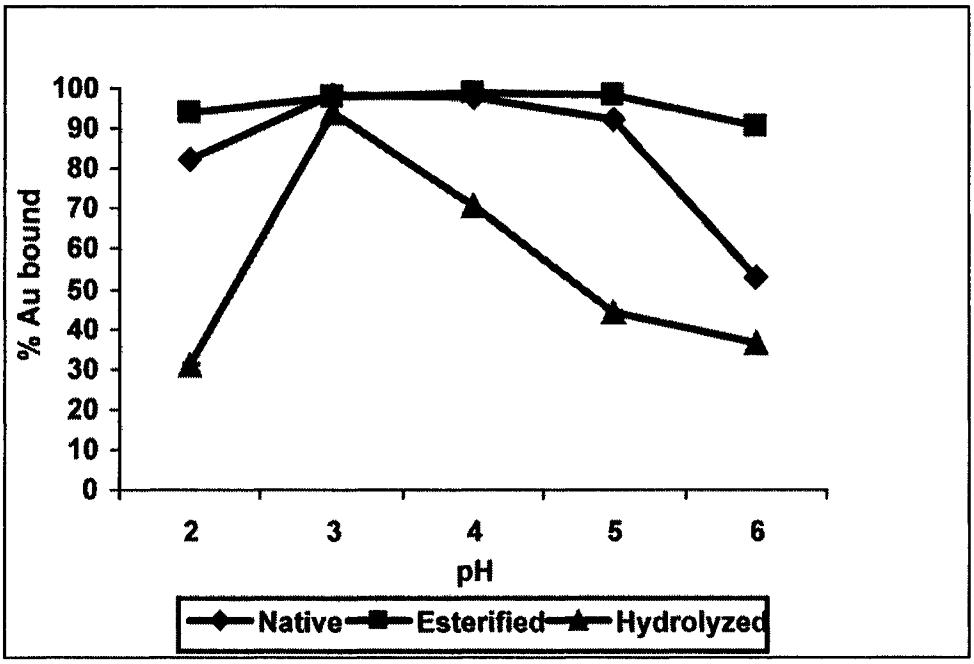

**Fig. 1:** pH profile for Au(III) binding to native, esterified and hydrolyzed hops biomass. Biomasses were allowed to react for 1 hour with a 0.1mM Au(III) solution.

binding. In addition, protonated amino and sulfhydryl groups may be able to interact with the Au anion participating in the binding of the metal to the hops biomass.

### Time dependency studies for Au (III) binding to hops biomass

Time dependency studies were performed at pH 2 and 5 in order to determine the optimum time for Au(III) binding to native, esterified, and hydrolyzed cone hops biomasses. Figure 2 shows that at pH 2, Au(III) binds very quickly to the three biomasses but in a different percentage. Au(III) bound better to the esterified (almost 100% of the amount of gold present in the solution) than to native (65%) or hydrolyzed (30%) biomasses. As shown in Figure 2, the binding is time independent since in every case the percentage of Au(III) bound remained the same for up to 60 min. The results obtained are comparable to those reported by Gamez et al. /15/, who found that the binding of Au(III) to alfalfa shoots biomass at pH 2 occurs rapidly and remains stable for up to 60 minutes.

Figure 3 shows the results for time dependency studies performed at pH 5 using native, esterified, and hydrolyzed cone hops biomasses. Contrary to the results obtained at pH 2, Figure 3 shows that at pH 5, Au(III) binding to the three biomasses was time dependent, showing the maximum binding at 60 min of reaction with a 0.1mM Au(III) solution. This may be attributed to the fact that at pH 5 all the groups are deprotonated and there are more negative charges decreasing the binding of the Au(III) anion to the hops biomasses. However, the three biomasses behaved differently as the reaction time progressed. As one can see in Figure 3, the maximum Au(III) binding to esterified hops biomass occurred during the first 10 minutes of

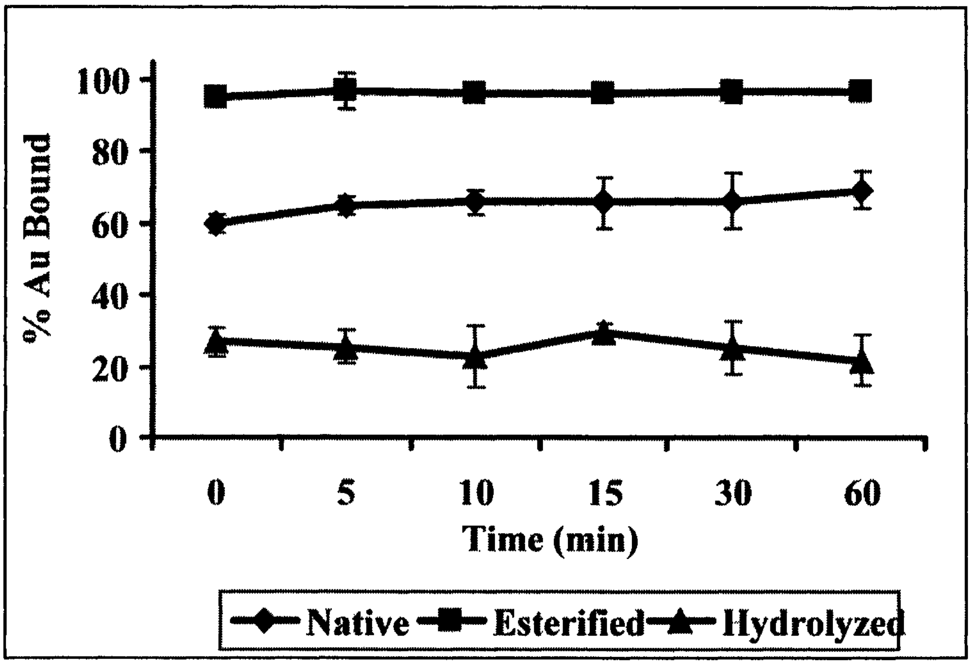

**Fig. 2:** Time dependence studies for native, esterified and hydrolyzed hops biomass at pH 2. Experiments were performed using a 0.1mM Au(III) solution.

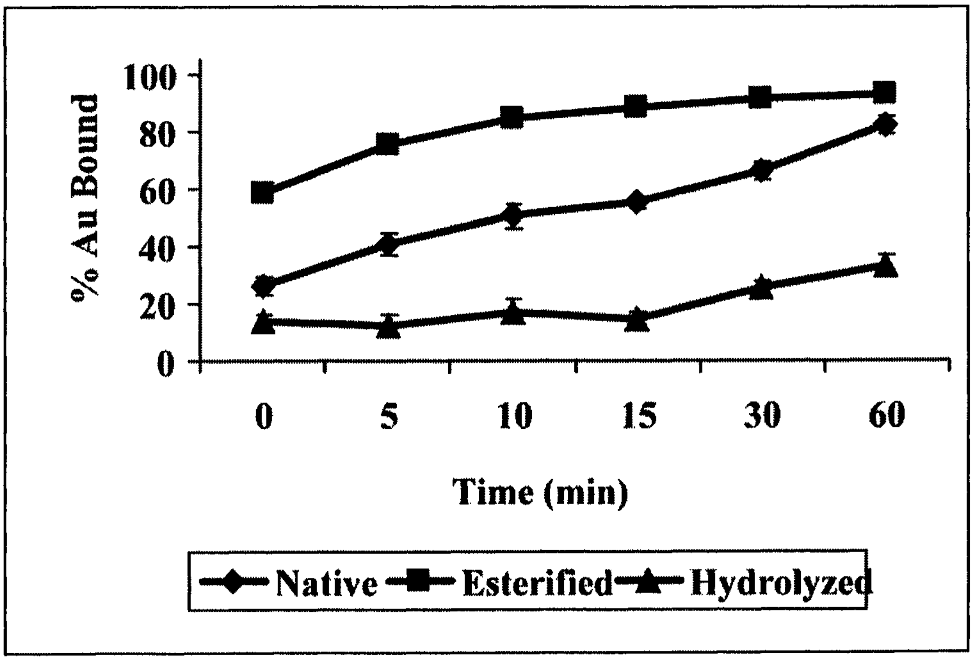

**Fig. 3:** Time dependence studies for native, esterified and hydrolyzed hops biomass at pH 5. Experiments were performed using a 0.1mM Au(III) solution.

the reaction. For the hydrolyzed hop biomass the binding started to occur after 15 minutes, but native hops biomass bound Au(III) ions linearly. Presumably, this divergent behavior of the biomasses is due to the chemical modifications, which produces different available groups for Au(III) binding. Au(III) and Au(I) are classified as a soft acids, this implies that gold ions form complexes with soft bases as nitrogen and sulfur (due to the lone pair of electrons). In addition, at pH 5, the amino groups of the hops biomass should be protonated and they could be involved in an electrostatic attraction to the gold anion /18/. We hypothesize that this could also be due to the fact that at pH 5, there is some Au(III) reduction.

### Au(III) binding capacity experiments

The results for the binding capacity studies in native and chemically modified cone hops biomasses at pH 2 are given in Table 1. The saturation of the biomasses was carried out after 9 Au binding cycles with a 0.3mM Au(III) solution. This table shows that the native and esterified biomasses exhibited a binding capacity close to 30 mg of Au per gram of biomass. On the other hand, the binding capacity of the hydrolyzed hop biomass was about 12 mg Au per gram of biomass. Thus, at pH 2, native and esterified hops biomasses showed a better Au(III) binding capacity as compared to the one exhibited by the hydrolyzed hops biomass.

**Table 1**

Capacities for Au(III) binding to native and chemically modified hops biomasses at pH 2[a].

| Biomass | Capacity (mg metal/g biomass) |
|---|---|
| Native | 28.24 ± 1.62 |
| Esterified | 33.40 ± 2.25 |
| Hydrolyzed | 12.04 ± 2.17 |

[a]Experiments were performed with a 0.3mM Au(III) solution and 9 saturation cycles. Each treatment was replicated three times for QC/QA purposes. Numbers are average ± 95% confidence interval (n=3).

A decrease in negative charges due to esterification may account for the increasing of Au(III) binding to biomass and may also suggest that sulfhydryl and amino groups are involved in the binding process as previously described /15/. Gamez *et al.* /15/ reported that Au(III) binding to esterified algal biomass increased when compared to native and hydrolyzed algae biomasses. Hydrolization of biomass involves an increase of negative charges on binding sites and consequently, an impediment for Au(III) binding to the biomaterial /17/. This may indicate that carboxyl groups are not mainly responsible for the binding of Au(III) to hops biomass. The amino and sulfhydryl groups mentioned before may also be involved in Au(III) binding to hops biomass.

In the present investigation, all biomasses presented a purple color within a period of one hour of reaction with the 0.3mM Au(III) solution. It was also observed that the purple color on the esterified biomass was more intense as compared to the other biomasses. The purple color has also been observed in alfalfa biomass and it is attributed to the reduction of Au(III) to Au(0) /15/. The difference in the intensity indicates the degree of reduction and may also point out that native and esterified hops biomasses have chemical groups more likely to react with Au(III), thus allowing for more metal adsorption and reduction.

The amounts of Au(III) bound to native and chemically modified cone hops biomasses at pH 5 are shown in Table 2. Comparing the data of Table 1 and Table 2, it is apparent that the capacities of the hydrolyzed hops biomass remained constant at pH 2 and 5 (around 12 mg of Au/g). However, there is a 30% increase on the Au(III) binding to the native hops biomass and about 10% increase on the Au(III) binding to the esterified hops biomass at pH 5. Perhaps, the increments on Au binding to native and esterified hops biomasses at this pH conform to a .fast reduction process that facilitates more binding sites within the biomass.

**Table 2**

Capacities for Au(III) binding to native and chemically modified hops biomasses at pH 5[a].

| Biomass | Capacity (mg metal/g biomass) |
|---|---|
| Native | 38.86 ± 2.35 |
| Esterified | 37.80 ± 1.55 |
| Hydrolyzed | 11.38 ± 1.00 |

[a]Experiments were performed with a 0.3mM Au(III) solution and 9 saturation cycles. Each treatment was replicated three times for QC/QA purposes. Numbers are average ± 95% confidence interval (n=3).

### Stripping of gold

It is important to determine if the gold bound to the hops biomass can be recovered. The stripping of gold was performed by exposing the gold laden biomass to different concentrations of thiourea at pH 2 and pH 5 during 3 cycles, following the procedure described in the literature /17/. Thiourea has been employed as a leaching agent (complexing agent) that forms a complex with gold. Figures 4 and 5 show the percentage of Au(III) recovered using different solutions of thiourea at pH 2 and pH 5. The concentrations of thiourea used in these experiments were 0.1M, 0.2M, 0.4M, and 0.6M. As shown in these figures, the highest percentage of Au(III) recovered was observed at pH 5. This suggests that Au(III) could be bound more strongly to native and modified hops biomasses at pH 2 than at pH 5, and consequently it is more difficult to be desorbed. Also, the complexation reaction could be pH dependent.

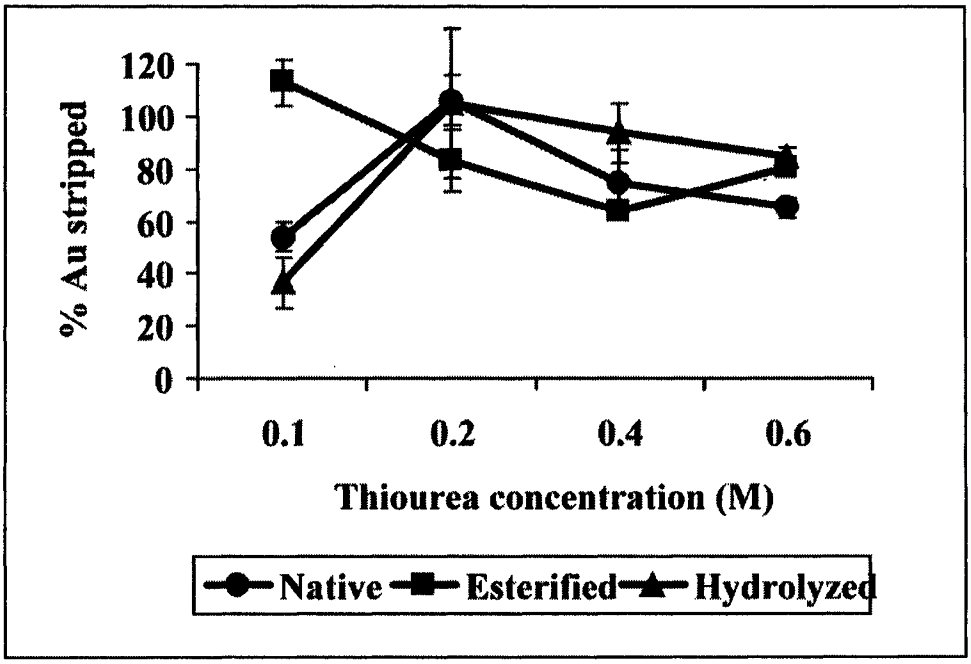

**Fig. 4:** Recovery of Au from native, esterified and hydrolyzed hops biomass using different concentrations of thiourea adjusted to pH 2.

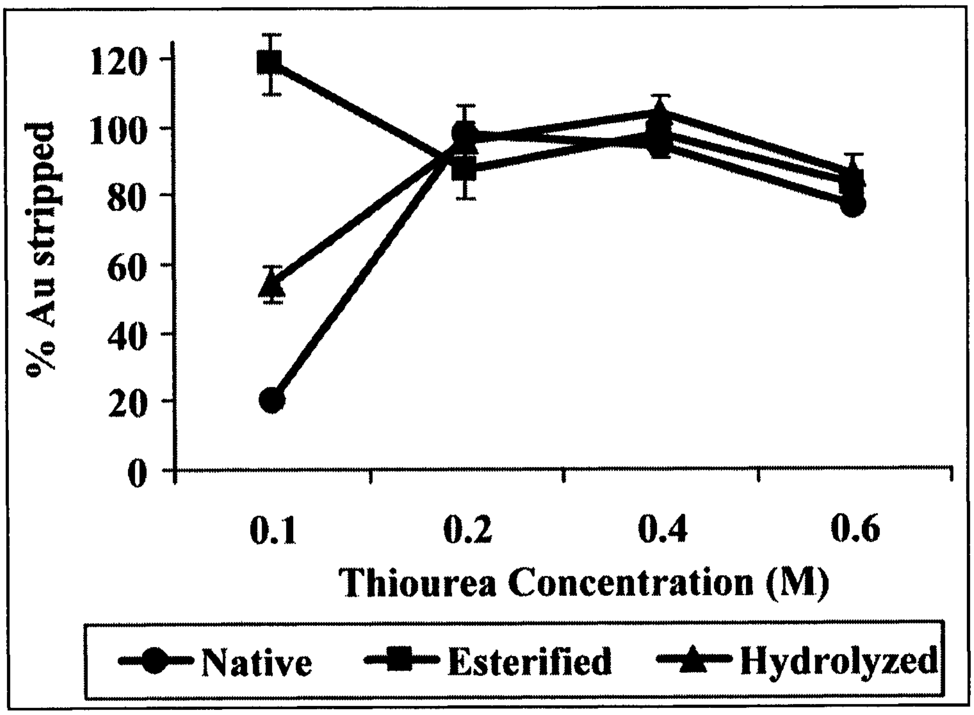

**Fig. 5:** Recovery of Au from native, esterified and hydrolyzed hops biomass using different concentrations of thiourea adjusted to pH 5.

### CONCLUSIONS

In summary, our results showed that cone hops biomass was able to bind Au(III) from an aqueous solution in a pH dependent manner. Time dependence studies demonstrated that Au(III) binding to the native hops biomass was time independent at pH 2 while at pH 5 it was time dependent. Capacity studies for native and esterified hops biomasses revealed a higher capacity for binding Au(III) ions from aqueous solutions at pH 2 and pH 5 as compared to hydrolyzed hops biomass. Also, stripping of gold was best performed using 0.2 M thiourea. The results obtained demonstrated that the cone hops biomass is effective in removing gold from aqueous solutions and could be considered a potential cost-effective and environmentally safe method for the recovery of gold.

### ACKNOWLEDGEMENTS

The authors acknowledge the support of the National Institutes of Health (grant S06GM8012-33) and the University of Texas at El Paso's Center for Environmental Resource Management (CERM) through funding from the Office of Exploratory Research of the U.S. Environmental Protection Agency (cooperative agreement CR-819849-01). The authors also thank the U.S. Department of Energy (DOE) Grant DEFC0401AL67097, Southwest Border and Technology Collaboration Program, and the Materials Corridor Initiative. We also thank the HBCU/MI Environmental Technology Consortium, that is funded by the Department of Energy. Dr. Gardea-Torresdey acknowledges the funding from National Institute of Environmental Health Sciences (Grant R01ES11367-01).

### REFERENCES

1. P. Gomes, M.F. Almeida, J.M. Loureiro, *Sep. Purif. Technol,* **24,** 35-57 (2001).
2. G.L. Miltzarek, C.H. Sampaio, J.L. Cortina, *Miner. Eng,* **15,** 75-82 (2002).
3. M.M. Figueira, B. Volesky, H.J. Mathieu, *Environ. Sci. Technol,* **33,** 1840-1846 (1999).
4. M. Zhao, J.R. Duncan, *Biotechnol. Lett,* **19**(10), 953-955 (1997).
5. H. Seki, A. Susuki, *J. Colloid Interface Sci,* **249,** 295-300 (2002).
6. R.S. Bai, T.E. Abraham, *Water Res,* **36,** 1224-1236 (2002).
7. J.G. Parsons, G. Gamez, K.J. Tiemann, J.L. Gardea-Torresdey, *Proceedings of the 2000 Conference on Hazardous Waste Research,* (2001). Edited by L.E. Erickson, M.M. Rankin, Kansas State Univ., and Manhattan, KS. pp 2-12.
8. J.L. Gardea-Torresdey, K.J. Tiemann, G. Gamez, K. Dokken, I. Cano-Aguilera, L.R. Furenlid, M.W. Renner, *Environ. Sci. Technol,* **34,** 4392-4396 (2000).
9. D. Kratochvil, B. Volesky, *Wat. Res,* **34,** 3186-3196 (2000).
10. D.W. Darnall, B. Greene, M.T. Henzl, J.M. Hosea, A. Robert, J. Sneddon, M.A. Dale, *Environ. Sci. Technol,* **20,** 206-208 (1986).

11. Y. Goksungur, S. Uren, U. Guvenc, *Turk. J. Biol,* **27,** 23-29 (2003).
12. H. Niu, B. Volesky, *J. Chem. Technol. Biotechnol,* **75,** 436-442 (2000).
13. C. Garbizu, I. Alkorta, *Bioresource Technol,* **77**(3), 229-236 (2001).
14. J.L. Gardea-Torresdey, K.J. Tiemann, G. Gamez, K. Dokken, N.E. Pingitore, *Adv. Environ. Res,* **3**(1), 83-93 (1999).
15. G. Gamez, J.L. Gardea-Torresdey, K.J. Tiemann, J. Parsons, K. Dokken, M. Jose-Yacaman, *Adv. Environ. Res,* 7(2), 563-571 (2003).
16. J.L. Gardea-Torresdey, M.K. Becker-Hapak, J.M. Hosea, D.W. Darnall, *Environ. Sci. Technol,* **24**(9), 1372-1378 (1990).
17. J.L. Gardea-Torresdey, K.T. Tiemann, K. Dokken, G. Gamez, *Proceedings of the 1998 Conference on Hazardous Waste Research* 111-121 (1998).
18. J.L. Gardea-Torresdey, K.J. Tiemann, J.G. Parsons, G. Gamez, I. Herrera, M. Jose-Yacaman, *Microchem. J,* **71,** 193-204 (2002).

